# Language impairment in adults with end-stage liver disease: application of natural language processing towards patient-generated health records

**DOI:** 10.1038/s41746-019-0179-9

**Published:** 2019-11-04

**Authors:** Lindsay K. Dickerson, Masoud Rouhizadeh, Yelena Korotkaya, Mary Grace Bowring, Allan B. Massie, Mara A. McAdams-Demarco, Dorry L. Segev, Alicia Cannon, Anthony L. Guerrerio, Po-Hung Chen, Benjamin N. Philosophe, Douglas B. Mogul

**Affiliations:** 1 Johns Hopkins University School of Medicine, Baltimore, MD USA; 2 Institute for Clinical and Translational Research, Johns Hopkins University School of Medicine, Baltimore, MD USA; 3 Department of Pediatrics, Johns Hopkins University School of Medicine, Baltimore, MD USA; 4 Department of Epidemiology, Johns Hopkins University Bloomberg School of Public Health, Baltimore, MD USA; 5 Department of Surgery, Johns Hopkins University School of Medicine, Baltimore, MD USA; 6 Kennedy Krieger Institute, Baltimore, MD USA; 7 Department of Internal Medicine, Johns Hopkins University School of Medicine, Baltimore, MD USA

**Keywords:** Liver diseases, Neurological manifestations, Machine learning, Language

## Abstract

End-stage liver disease (ESLD) is associated with cognitive impairment ranging from subtle alterations in attention to overt hepatic encephalopathy that resolves after transplant. Natural language processing (NLP) may provide a useful method to assess cognitive status in this population. We identified 81 liver transplant recipients with ESLD (4/2013–2/2018) who sent at least one patient-to-provider electronic message pre-transplant and post-transplant, and matched them 1:1 to “healthy” controls—who had similar disease, but had not been evaluated for liver transplant—by age, gender, race/ethnicity, and liver disease. Messages written by patients pre-transplant and post-transplant and controls was compared across 19 NLP measures using paired Wilcoxon signed-rank tests. While there was no difference overall in word length, patients with Model for End-Stage Liver Disease Score (MELD) ≥ 30 (*n* = 31) had decreased word length in pre-transplant messages (3.95 [interquartile range (IQR) 3.79, 4.14]) compared to post-transplant (4.13 [3.96, 4.28], *p* = 0.01) and controls (4.2 [4.0, 4.4], *p* = 0.01); there was no difference between post-transplant and controls (*p* = 0.4). Patients with MELD ≥ 30 had fewer 6+ letter words in pre-transplant messages (19.5% [16.4, 25.9] compared to post-transplant (23.4% [20.0, 26.7] *p* = 0.02) and controls (25.0% [19.2, 29.4]; *p* = 0.01). Overall, patients had increased sentence length pre-transplant (12.0 [9.8, 13.7]) compared to post-transplant (11.0 [9.2, 13.3]; *p* = 0.046); the same was seen for MELD ≥ 30 (12.3 [9.8, 13.7] pre-transplant vs. 10.8 [9.6, 13.0] post-transplant; *p* = 0.050). Application of NLP to patient-generated messages identified language differences—longer sentences with shorter words—that resolved after transplant. NLP may provide opportunities to detect cognitive impairment in ESLD.

## Introduction

End-stage liver disease (ESLD) is associated with a wide spectrum of neurocognitive impairment ranging from minimal alterations in attention, working memory, and psychomotor speed to coma and death.^1–3^ Approximately 80% of patients with ESLD have neurocognitive changes associated with poorer quality of life, including deteriorating sleep and work performance. As many as 20% of adults with ESLD can develop the most severe form of cognitive impairment, overt hepatic encephalopathy (HE), which may portend up to 43% mortality at one year.^1,3–6^

Despite the considerable burden caused by these cognitive deficits, diagnosis remains challenging. Common diagnostic modalities include blood tests (e.g., ammonia, amino acid profiles), neurocognitive assessments (e.g., Wechsler Adult Intelligence Scale-Fourth Edition, Delis–Kaplan Executive Function System, The Stroop Color and Word Test), computer-based test batteries, electroencephalogram, and imaging.^1,7–12^ While the Psychometric HE Score (PHES), a paper–pencil test battery, is considered to be the gold standard for diagnosis of minimal HE (MHE), it remains labor-intensive to administer and requires an in-person clinical visit.^10^ Other modalities such as blood ammonia levels correlate poorly with neurocognitive testing and severity of HE.^13,14^ Given the absence of precise diagnosis and substantial mortality risk associated with late-stage encephalopathy, there exists a need for a real-time and determinate means to identify ESLD-related cognitive impairment.

Research on potential language impairment in ESLD is limited and inconclusive.^15^ One study demonstrated that patients with MHE had deficits in verbal fluency and phrase construction that resolved after transplantation, while other research has concluded that language remains largely intact.^16,17^ Techniques developed from the field of natural language processing (NLP)—a subfield of computer science employing computational techniques to learn, understand, and produce human language content—offer an innovative approach for evaluating ESLD-related language alterations.^18^ NLP is increasingly common in non-medical contexts, with its myriad uses including virtual voice-based assistants (e.g. Siri from Apple, Alexa from Amazon), therapy chat bots (e.g. Woebot), and forensic linguistics methods to identify authors of anonymous publications.^19^ Despite the widespread utilization of electronic medical records (EMRs), application of NLP technology has only recently extended to healthcare and is typically exploited more often for analyzing provider documentation than assessing patients’ written text or transcribed spoken language. Examples include: identification of key words in patients’ electronic notes that indicate bleeding, use in an algorithm to stratify risk in patients with cirrhosis, and incorporation into a mortality prediction model in the intensive care unit (ICU).^20–22^

The aim of this pilot study was to determine if NLP-based methods can identify and characterize language differences in patients with ESLD that may suggest the presence of neurocognitive impairment. Thus, we evaluated and compared the language patterns in patient-generated electronic messages written before and after transplant and by “healthy” controls with liver disease but who were not undergoing evaluation for transplantation. We hypothesized that NLP would detect language alterations that resolved after transplantation, with the post-transplant messages being similar to the language patterns of controls.

## Results

### Study population

The median age (interquartile range, IQR) of the 81 transplanted patients included in analysis was 53.8 years (19–69; Table 1). Among these, 40% were female, 73% were Caucasian, and 38% had a Model for End-stage Liver Disease (MELD) score ≥30 at transplant; controls matched demographically. The most frequent indication for transplant was hepatitis C virus (HCV) cirrhosis (33.3%), followed by nonalcoholic steatohepatitis (NASH) cirrhosis (17.3%). The median (IQR) number of messages per patient was 3 (1–10) in the pre-transplant period, 10 (4–24) post-transplant, and 3 (2–5) for controls. For patients with MELD ≥ 30, the median number of messages per patient was 5 (1–18) in the pre-transplant period, 15 (5–39) post-transplant, and 2 (1–4) for controls. A transjugular Intrahepatic portosystemic shunt was placed in 2% of cases and no controls. Among cases, 54% people used at least one drug that can treat HE, including 16% that used rifaximin, 7% that used lactulose, and 25% that used both. In contrast, 1% of controls (*n* = 1) used rifaximin and it was prescribed for bacterial overgrowth. Though cases and controls were not matched by use of drugs that may influence cognition (e.g., opioids), the frequency of their use was similar in each group.

**Table 1:** Demographics, Model for End-stage Liver Disease (MELD) score, message number, and transplant indication

Characteristic	Controls	Overall	MELD ≥ 30
	*n* = 81	*n* = 81	*n* = 31
Age (years)	Mean (range)	Mean (range)	Mean (range)
	53.7 (20–70)	53.8 (19–69)	52.6 (30–69)
Gender	*n* (%)	*n* (%)	*n* (%)
Female	32 (40)	32 (40)	15 (48.4)
Race and ethnicity	*n* (%)	*n* (%)	*n* (%)
Asian	2 (2.5)	2 (2.5)	1 (3.2)
Black/African American	14 (17.3)	14 (17.3)	8 (25.8)
Hispanic	2 (2.4)	1 (1.2)	0 (0.0)
Mixed/Other	5 (6.2)	5 (6.2)	2 (6.5)
White/Caucasian (non-Hispanic)	58 (71.6)	59 (72.8)	20 (64.5)
Pre-transplant MELD	–	*n* (%)	*n* (%)
≤9	–	5 (6.2)	–
10–19	–	11 (13.6)	–
20–29	–	34 (41.9)	–
30–39	–	23 (28.4)	23 (74.2)
≥40	–	8 (9.9)	8 (25.8)
Message number	Median (IQR)	Median (IQR)	Median (IQR)
Pre-transplant	3 (2–5)	3 (1–10)	5 (1–18)
Post-transplant	–	10 (4–24)	15 (5–39)
Transplant indications	*n* (%)	*n* (%)	*n* (%)
Hepatitis C virus (HCV) cirrhosis	26 (32.1)	27 (33.3)	8 (25.8)
Nonalcoholic steatohepatitis (NASH) cirrhosis	14 (17.3)	14 (17.3)	4 (12.9)
Primary sclerosing cholangitis	9 (11.1)	12 (14.8)	10 (32.3)
Alcoholic cirrhosis	3 (3.7)	11 (13.6)	4 (12.9)
Abnormal LFTs	16 (19.8)	–	–
Other	13 (16.0)	17 (21)	5 (16.1)
Use of drugs that influence cognition	*n* (%)	*n* (%)	
Narcotics	23 (28%)	18 (22%)	
Antiepileptics	4 (5%)	3 (4%)	
Antipsychotics	1 (1%)	3 (4%)	
Anxieolytics	17 (21%)	14 (17%)	
Other	2 (2%)	2 (2%)	

### Lexical domain

#### Word length

Among all patients with ESLD (*n* = 81), word length was similar pre-transplant (median [IQR]: 4.06 [3.85, 4.35]) and post-transplant (4.12 [3.99, 4.23]; *p* = 0.9; Table 2). Word length in control messages (4.1 [3.9, 4.4]) was also similar to pre-transplant (*p* = 0.6) and post-transplant (*p* = 1.0). Among patients with MELD ≥ 30, words were on average shorter in pre-transplant messages (3.95 [3.79, 4.14]) compared to post-transplant (4.13 [3.96, 4.28]; *p* = 0.01) and controls (4.2 [4.0, 4.4]; *p* = 0.01) (Table 3). There was no difference in word length between post-transplant and control messages for MELD ≥ 30 (*p* = 0.4).

**Table 2:** Change in NLP measures between messages pre-transplant to post-transplant and vs. controls

NLP measure	Pre-transplant	Post-transplant	*p*-value	Controls	*p*-value^a^	*p*-value^b^
	Median (IQR)	Median (IQR)		Median (IQR)		
Lexical						
Word length	4.06 (3.85, 4.35)	4.12 (3.99, 4.23)	0.9	4.1 (3.9, 4.4)	0.6	1
6+ letter words (%)	23.4 (18.7, 27.3)	23.0 (20.2, 25.0)	0.6	22.8 (18.6, 27.3)	0.6	0.8
Numeral words (%)	2.2 (0.8, 4.0)	1.8 (0.9, 3.2)	0.1	1.5 (0, 2.9)	0.1	0.6
Capitalized words (%)	7.1 (3.2, 11.2)	7.2 (3.5, 13.8)	0.5	6.0 (3.1, 11.8)	0.9	0.5
Lexico-syntactic						
Pronouns (%)	11.5 (9.7, 14.5)	12.0 (10.1, 13.2)	0.9	13.4 (11.1, 15.4)	0.048	0.02
Function words (%)	36.2 (32.0, 39.0)	35.2 (31.6, 37.5)	0.3	36.6 (32.0, 39.2)	0.9	0.3
Question words (%)	0.6 (0.0, 1.3)	0.6 (0.3, 1.0)	0.9	0.6 (0, 1.2)	0.8	0.8
Verbs (%)	17.3 (15.2, 20.8)	17.1 (16.0, 18.7)	0.4	17.3 (14.8, 18.8)	0.3	0.6
Adjectives (%)	4.2 (2.9, 5.9)	4.8 (3.7, 5.8)	0.2	4.6 (2.7, 6.2)	0.2	0.9
Nouns (%)	22.1 (20.0, 26.1)	23.0 (20.7, 26.0)	0.8	21.7 (18.5, 26.0)	0.6	0.2
Syntactic						
Sentence length	12.0 (9.8, 13.7)	11.0 (9.2, 13.3)	0.046	10.5 (8.7, 13.8)	0.1	0.5
Noun phrase ratio	1.59 (1.49, 1.75)	1.59 (1.51, 1.73)	0.2	1.6 (1.4, 1.7)	0.1	0.5
Subj–verb–object ratio	6.3 (4.6, 8.1)	6.2 (4.9, 7.2)	0.3	5.2 (3.7, 7.6)	0.1	0.2
Flesch–Kincaid grade	4.4 (2.9, 5.0)	4.0 (2.8, 5.2)	0.3	4.2 (2.9, 5.2)	0.5	0.6
Lexico-semantic						
Unique words (%)	85.5 (80.0, 89.9)	85.6 (80.3, 89.2)	0.8	85.1 (80.5, 90.2)	0.8	0.8
Brunét’s Index	7.3 (6.6, 8.2)	7.3 (6.4, 8.4)	0.9	7.3 (6.2, 8.2)	0.2	0.3
Honoré’s statistic	2242.4 (2034.6, 2644.8)	2347.6 (2055.1, 2681.2)	0.9	2175.9 (1863.6, 2573.2)	0.3	0.2
Sentiment						
Polarity	0.04 (0.00, 0.10)	0.06 (0.02, 0.11)	0.1	0.05 (0.01, 0.10)	0.2	0.7
Subjectivity	0.18 (0.11, 0.24)	0.19 (0.15, 0.24)	0.2	0.18 (0.09, 0.25)	0.7	0.3

^a^Pre-transplant vs. controls

^b^Post-transplant vs. controls

**Table 3:** Change in NLP measures among patients w/MELD ≥ 30 at transplant (*n* = 31)

NLP measure: median (IQR)	Pre-transplant	Post-transplant	*p*-value	Controls	*p*-value^a^	*p*-value^b^
	Median (IQR)	Median (IQR)		Median (IQR)		
Lexical						
Word length	3.95 (3.79, 4.14)	4.13 (3.96, 4.28)	0.01	4.2 (4.0, 4.4)	0.01	0.4
6+ letter words (%)	19.5 (16.4, 25.9)	23.4 (20.0, 26.7)	0.02	25.0 (19.2, 29.4)	0.01	0.6
Numeral words (%)	1.7 (0.4, 5.5)	1.8 (0.8, 2.2)	0.2	1.3 (0, 3.1)	0.5	0.8
Capitalized words (%)	8.9 (3.9, 11.2)	8.8 (5.2, 14.3)	0.7	6.1 (3.7, 9.1)	0.3	0.4
Lexico-syntactic						
Pronouns (%)	11.5 (8.9, 14.1)	12.1 (10.3, 13.1)	0.3	13.5 (11.9, 15.4)	0.02	0.1
Function words (%)	36.2 (32.0, 40.1)	34.9 (31.8, 37.1)	0.4	37.4 (33.3, 41.7)	0.4	0.2
Question words (%)	0.7 (0.3, 1.5)	0.7 (0.3, 1.2)	0.4	0.4 (0, 0.8)	0.1	0.1
Verbs (%)	17.4 (16.3, 21.0)	17.7 (16.3, 18.7)	0.6	17.4 (15.7, 19.8)	0.8	0.8
Adjectives (%)	4.2 (2.7, 5.9)	4.5 (3.0, 5.7)	0.3	5.2 (2.4, 6.5)	0.2	0.7
Nouns (%)	21.4 (17.5, 25.0)	23.3 (19.7, 26.5)	0.2	22.2 (17.9, 26.8)	0.8	0.2
Syntactic						
Sentence length	12.3 (9.8, 13.7)	10.8 (9.6, 13.0)	0.05	10.8 (8.6, 15.1)	0.3	0.9
Noun phrase ratio	1.63 (1.47, 1.82)	1.55 (1.45, 1.71)	0.03	1.6 (1.5, 1.7)	0.4	0.2
Subj–verb–object ratio	6.5 (5.6, 8.7)	6.9 (6.1, 8.3)	0.7	6.8 (4.1–9.1)	0.8	0.7
Flesch–Kincaid grade	3.9 (2.8, 5.0)	4.2 (3.3, 4.7)	0.3	4.4 (3.3, 5.7)	0.4	0.5
Lexico-semantic						
Unique words (%)	87.1 (81.6, 93.9)	86.2 (82.7, 90.6)	0.8	85.2 (81.1–87.8)	0.5	0.5
Brunét’s Index	7.1 (6.4, 8.0)	7.2 (6.2, 7.6)	0.8	7.6 (5.6, 8.1)	0.8	0.7
Honoré’s statistic	2388.7 (2115.8, 2844.6)	2398.7 (2130.3, 2713.2)	0.8	2197.2 (1937.2, 2583.2)	0.2	0.4
Sentiment						
Polarity	0.05 (0.02, 0.10)	0.05 (0.02, 0.09)	1	0.04 (0.01, 0.11)	0.4	0.7
Subjectivity	0.19 (0.11, 0.26)	0.19 (0.15, 0.22)	0.7	0.18 (0.09, 0.28)	0.7	0.8

^a^Pre-transplant vs. controls

^b^Post-transplant vs. controls

#### 6+ letter words

Among all patients with ESLD, there were no differences in the percent of 6+ letter words pre-transplant compared to post-transplant or controls. However, patients with MELD ≥ 30 had a lower percentage of 6+ letter words in pre-transplant messages (19.5 [16.4, 25.9] compared to post-transplant (23.4 [20.0, 26.7]; *p* = 0.02) and controls (25.0 [19.2, 29.4]; *p* = 0.01). There was no difference in percent of 6+ letter words between post-transplant and control messages for MELD ≥ 30 (*p* = 0.6).

#### Numeral words, capitalized words

There were no differences in the percent of numeral words or capitalized words between pre-transplant and post-transplant messages or control messages, overall or for those with MELD ≥ 30.

### Lexico-syntactic domain

#### Pronouns

Among all patients with ESLD, there was no difference in percent of pronouns between pre-transplant (11.5 [9.7, 14.5]) and post-transplant messages (12.0 [10.1, 13.2]; *p* = 0.9). Control messages contained a higher percent of pronouns (13.4 [11.1, 15.4]) when compared to pre-transplant (*p* = 0.048) and post-transplant (*p* = 0.02). Among patients with MELD ≥ 30, there was no difference in percent of pronouns between pre-transplant (11.5 [8.9, 14.1] and post-transplant messages (12.1 [10.3, 13.1]; *p* = 0.3). However, patients with MELD ≥ 30 had a lower percent of pronouns in pre-transplant messages compared to controls (13.5 [11.9, 15.4]; *p* = 0.02), and similar percent of pronouns in post-transplant and control messages (*p* = 0.1).

#### Function words, question words, verbs, adjectives, nouns

Among all patients with ESLD and those with MELD ≥ 30, there were no differences in these NLP measures between pre-transplant, post-transplant, and control messages.

### Syntactic domain

#### Sentence length

Among all patients with ESLD, sentence length was increased in pre-transplant messages (12.0 [9.8, 13.7]) compared to post-transplant (11.0 [9.2, 13.3]; *p* = 0.046). Control messages had slightly decreased sentence length (10.5 [8.7, 13.8]), however, this was not statistically significant when compared to pre-transplant (*p* = 0.1) or post-transplant (*p* = 0.5) messages. Patients with MELD ≥ 30 had marginally increased sentence length in pre-transplant messages (12.3 [9.8, 13.7]) when compared to post-transplant (10.8 [9.6, 13.0]; *p* = 0.050). Control messages had slightly decreased sentence length (10.8 [8.6, 15.1]), however, this was not statistically significant when compared to pre-transplant (*p* = 0.3) or post-transplant (*p* = 0.9) messages.

#### Noun phrase ratio

Among all patients with ESLD, there was no difference in the noun phrase ratio pre-transplant and post-transplant, or when compared to controls. Among patients with MELD ≥ 30, the noun phrase ratio was greater in pre-transplant (1.63 [1.47, 1.82]) than in post-transplant messages (1.55 [1.45, 1.71; *p* = 0.03). However, there were no differences between control messages (1.6 [1.5, 1.7]) and pre-transplant (*p* = 0.4) or post-transplant (*p* = 0.2) among those with MELD ≥ 30.

#### Subject–verb–object ratio, Flesch–Kincaid grade readability score

Among all patients with ESLD and those with MELD ≥ 30, there were no differences in subject–verb–object ratio or Flesch–Kincaid grade readability score between pre- and post-transplant messages or control messages.

### Lexico-semantic domain

#### Unique words, Brunét’s Index, Honoré’s statistic

Among all patients with ESLD and those with MELD ≥ 30, there were no differences observed in these lexico-semantic measures between pre-transplant, post-transplant, and control messages.

### Sentiment domain

#### Polarity, subjectivity

Among all patients with ESLD and those with MELD ≥ 30, there were no differences in polarity or subjectivity between groups.

## Discussion

In this retrospective, pilot study of patient-generated EMR messages, certain NLP measures identified linguistic differences in messages of adults with liver disease before as compared to after transplant. Most notably among patients with high MELD score, messages written in the pre-transplant period contained longer sentences consisting of shorter words and longer noun phrases, in contrast to shorter sentences with longer words and shorter noun phrases in the post-transplant period. After transplant, these differences largely resolved such that post-transplant messages were similar to controls. Thus, NLP identified differences primarily in the lexical and syntactic domains. Results were particularly pronounced in patients with MELD ≥ 30, indicating that NLP may be especially useful for identifying neurocognitive changes as liver disease severity worsens.

Although there are limited studies that have applied NLP tools to analyze cognitive impairment, to the best of our knowledge, this pilot study is the first analysis that applies these tools towards patient-generated EMR messages. Recently, Beltrami et al. compared recorded transcripts of people with known cognitive impairment and healthy controls and identified several lexical and syntactic differences between the groups.^15^ Presently about one quarter of people in the United States have engaged with their EMR, and about one in five people have sent a message to a provider, thus indicating the existence of troves of data that can be analyzed to better characterize changes in language in specific populations.^34^ If findings from our study can be replicated in larger populations, both with ESLD, as well as other disorders associated with cognitive deficits, it may represent a tremendous opportunity to identify screen for these disorders in as well as monitor their disease as it progresses.

While ESLD is known to be associated with neurologic and psychiatric complications, few studies have investigated the impact of ESLD on language. In one study by Mooney et al. using a test battery to examine cognitive dysfunction in patients with ESLD, language appeared preserved.^17^ In contrast, Adekanle et al. reported that patients with cirrhosis scored lower than controls in a test’s language domain with respect to naming, comprehension, fluency, definition, and repetition.^35^ Furthermore, Mattarozzi et al. found that word fluency (i.e. the ability to form and express words necessary for normal social and occupational function) and phrase construction (i.e. the grammatical arrangement of words in a phrase) improved 6 months after liver transplant in adults with cirrhosis and MHE.^16^ Furthermore, in children with chronic liver disease, de-Paula et al. noted delays in language development in children awaiting transplantation compared to those who had undergone transplantation.^36^

In our study, the somewhat surprising finding that pre-transplant messages were actually longer than post-transplant messages may suggest that cognitive impairment leads to rambling sentences with simpler words prior to transplant, compared to more concise sentences with longer words after transplant and in control messages (Supplementary Table 2). For example, one patient wrote pre-transplant: “I was asked by my wife to request that when you send the fax to my disability insurance company that if you could include the following (word count: 26) [list of information to include]. Once I go back to work full time and be off work again I will have to wait an extended period of time before, and if I will receive any benefits (word count: 31).” The same patient wrote post-transplant: “I have scheduled a small vacation and will be out of town for 3 days (word count: 15). My wife wants to make sure this will be ok (word count: 10).”

Given that language alterations may be subtle in liver disease, technology from NLP could fill a current gap in diagnosis of MHE, and provide an opportunity for earlier and more consistent detection of linguistic differences indicative of cognitive decline. Using NLP-based methods is an appropriate choice in this context for multiple reasons. First, these methods have been used successfully for diagnostic and prognostic purposes in neurological and psychiatric disorders.^37^ For example, Elvevag et al. employed a technique in NLP called latent semantic analysis (LSA) to quantify incoherence, a complex and nonconcrete measure, in the transcribed speech of patients with schizophrenia.^38^ In addition, Beltrami et al. deemed NLP techniques promising for identifying cognitive decline in the transcribed speech of patients with preclinical dementia.^15^ Second, as discussed by Kreimeyer et al. NLP has been shown to be a suitable tool for analyzing and gathering data from unstructured free text such as that in the EMR.^39^ For example, Amazon Web Services’ new NLP service Comprehend Medical takes advantage of this capability for clinical decision support, revenue cycle management, and other tasks.^40^ Application of NLP to identify subclinical cognitive decline in the EMR messages of patients with ESLD builds on the above research.

Although these pilot data suggest the potential for NLP tools to identify important abnormalities in cognition, one important limitation of this study was that we were unable to correlate our results to validated measurements or clinical consequences of HE, such as neurocognitive testing or inability to perform activities of daily living, respectively. Indeed, the abnormalities we are detecting here may also be seen in other forms of dementia, such as uremic encephalopathy of end-stage kidney disease or Alzheimer’s disease. Future prospective studies should include standardized cognitive assessment that are most suggestive of HE to, among other aims, determine a threshold at which NLP can identify alterations in cognition. A second limitation is that pre-transplant and post-transplant messages were analyzed within a single specific time period rather than longitudinally. While we chose to analyze for this pilot study the period before and after transplant, since this interval is likely to show the biggest change in cognition, additional studies should incorporate NLP technology over the course of months to establish potential changes in language over time as patients progress to, and subsequently recover following, transplant. Studies could likewise correlate variations in language as people are receiving therapy for HE to determine if treatment influences language as detected using NLP.

Additional limitations concern the patients that were included in analysis. For example, it is possible that patients with the most severe language abnormalities were excluded from results as they needed a proxy to write to their provider. While this could indicate language differences among groups were underestimated, it also highlights the importance of confirming that the message author is the patient of interest, should NLP technology be incorporated into an automatic clinical decision support system in the future. Similarly, only a subset of patients transplanted during the study interval (81/234 or 35%) met the inclusion criteria of having at least one message in the pre-transplant and post-transplant periods and alcoholic cirrhosis was underrepresented in the study population (13.6%) as compared to the the group of people that were excluded (*n* = 151) due to lack of EMR messages (34.4%). These observations suggest the possibility that findings from our study might not be representative of the general population. It is worth noting that the matched analysis of our study, in which cases are both compared before and after transplant as well as to controls with similar diseases, strengthens the internal validity of the findings even as one should be cautious in extending the findings to other groups. At the same time, it is now understood that use of the internet and smartphones has reached “near saturation” such that individuals from all socioeconomic groups at least have access to platforms such as EPIC MyChart, thereby allowing for the possibility that our findings could be generalized to other groups.^41^

An important next step would be to use NLP tools to prospectively analyze patients’ language in comparison to a standardized neuropsychological testing measure or other established clinical or diagnostic indicators of neurocognitive impairment. Subsequent findings may allow for the creation of a clinical decision support system built into the EMR that automatically analyzes patient-to-provider messages, alerting providers when further evaluation of a patient’s cognitive status is warranted. The hope would be that this could change the care delivered to the patient, and potentially even justify advocacy for higher placement on the transplant waitlist.

As NLP use in medicine grows, careful consideration should be given to ensuring clinical effectiveness, seamless integration into care processes, and high-value application of the technology. We believe patient-generated, unstructured free text in the EMR is a largely untapped cache of data for which NLP analysis is uniquely suited. While NLP has shown promise in identifying cognitive impairment in patients with neurologic and psychiatric conditions through analysis of transcribed speech, the application of the technology to patient-generated EMR messages for the purpose of detecting language abnormalities is novel, particularly in evaluating ESLD-related cognitive decline.

## Methods

### Identification of transplanted patients

We identified 469 adults (>18 years) with ESLD who received a liver transplant at the Johns Hopkins Hospital (JHH) from April 1, 2013, when patient-generated electronic messages were incorporated into the EMR, to January 31, 2018. Participants were eligible if they had: (1) at least one patient-generated message in the “pre-transplant” period, defined as 6 months prior to the date of admission for transplant, (2) one patient-generated message in the “post-transplant” period, defined as between 30 days and 6 months after discharge, and (3) absence of an International Classification of Diseases, 10th revision (ICD-10) diagnosis code indicating a neurologic/psychiatric comorbidity or developmental disability (e.g. dementia, schizophrenia, or autism spectrum disorder; Supplemental Table 3). Among 469 transplanted patients, 205 were first excluded because they had an ICD-10 diagnosis code indicating neurologic/psychiatric comorbidity. Among the remaining 234 patients, 153 were excluded because they did not have at least one MyChart message in both the pre-transplant and post-transplant period. Eighty-one individuals with ESLD satisfied inclusion/exclusion criteria. The Institutional Review Board for each hospital in the Johns Hopkins Healthcare System approved this study. A waiver of informed consent was obtained by the Institutional Review Boards. The data are not publicly available given that they are not de-identifiable and could compromise patient privacy.

### Identification of healthy controls

Transplanted patients were matched 1:1 to “healthy” controls who had at least one patient-generated message based on the following criteria: age at transplant (±8 years), gender, and race/ethnicity, and diagnosis similar to the indication for transplant. For example, a patient who was transplanted for “alcoholic cirrhosis” was matched to a control with “alcoholic liver disease” and a patient transplanted for “hepatocellular carcinoma” was matched to a control with “benign neoplasm of the liver.” In instances in which no controls matched all the necessary characteristics, controls with the diagnosis of “abnormal liver function tests” were selected (Supplementary Table 3). All charts were manually reviewed to make sure potential controls had no evidence of cirrhosis as indicated by clinical notes, laboratory, imaging, or biopsy reports. Potential controls who had been referred for liver transplant evaluation or who had evidence of severe medical comorbidities (e.g., cancer, congestive heart failure), frequent hospitalizations, neurologic/psychiatric comorbidities, or developmental disability were excluded.

### Data extraction

The Johns Hopkins Healthcare System uses EPIC (Verona, WI), an EMR that allows for electronic messages to be sent through a portal called MyChart. The MyChart interface is similar to email, and patients access MyChart through a web-based portal on their computers, tablets, or smartphones. In addition to messages, demographic and clinical information was extracted including age, gender, race/ethnicity, indication for transplant, ICD-10 diagnosis codes, and allocation MELD score at transplant (i.e., score used for allocation, either the calculated score or the score with exception points, whichever is greater). A manual chart review was performed when a patient’s transplant indication was inconclusive based on ICD-10 codes. System-generated and redundant (i.e. copied) content was removed from the messages, and the text was segmented into individual sentences based on reasonable judgment of a native English-speaking reviewer (LD). Greetings, endings, and contact or identifying information, such as phone number, address, or date of birth was annotated and excluded from NLP results. Messages written by proxies, such as a spouse, were identified by the reviewer (LD) and removed from analysis.

### NLP-based measures

Nineteen NLP measures across five domains were analyzed using Python NLP Libraries, including Natural Language Toolkit and spaCy (Explosion AI, Berlin, Germany).

### Lexical domain

Lexical measures encompass the property of words and the vocabulary of language, and include word length (i.e. letters per word), percent of 6+ letter words, percent of numeral words, and percent of capitalized words.^23^

### Lexico-syntactic domain

Lexico-syntactic measures refer to the property of words in the context of a sentence’s grammatical structure (mainly parts of speech) and include percent of function words (i.e. conjunctions and prepositions such as “and,” “but,” “by,” “with”), question words, verbs, adjectives, nouns, and pronouns.^24^

### Syntactic domain

Syntactic measures characterize the grammatical structure of sentences and include sentence length (i.e. words per sentence), subject–verb–object ratio (i.e. number of subject–verb–object triples per sentence), noun phrase ratio (i.e. the sum of words in phrases functioning as the sentence’s subject, object, or prepositional object divided by the total number of these phrases), and Flesch–Kincaid grade level (i.e. the readability score of text expressed as a U.S. grade level based on calculations involving total words, sentences, and syllables).^25,26^

### Lexico-semantic domain

Lexico-semantic measures target the semantic use, or meaning, of words and lexical richness (i.e. the ratio of total words to unique words), such as percent of unique words (i.e. type–token ratio).^27^ Brunét’s Index (*W*), also in the lexico-semantic domain, generates a measure of lexical richness from calculations involving the total number of words (*N*) and the total vocabulary used (*V*), such that *W* = *N*^*v*−0.165^, with a lower value denoting richer text.^28,29^ Honore’s statistic (*R*) generates a lexical richness measure from calculations involving the total number of words, total vocabulary, and words used only once (*V*_1_), such that *R* = 100 log *N*/(1−*V*_1_/*V*), with a higher value denoting richer text.^30,31^

### Sentiment domain

Sentiment measures include polarity and subjectivity.^32,33^ Polarity quantifies the positive, negative, or neutral emotional content expressed in text while subjectivity quantifies the author’s personal feelings, opinion, or beliefs (polarity scale −1 to +1, subjectivity scale 0 to +1).

### Statistical analysis

NLP measures were calculated for each message. If patients sent multiple messages pre-transplant or post-transplant, then the average of the NLP measures was calculated to represent their pre-transplant or post-transplant results, respectively. Paired Wilcoxon signed-rank tests were used to compare the NLP measures in messages written by patients with ESLD pre-transplant versus post-transplant, pre-transplant versus “healthy” controls, and post-transplant versus healthy controls (*n* = 81). Similar analyses were performed for the subgroup of ESLD patients with MELD ≥ 30 (*n* = 31). All analyses were assessed for statistical significance at the *α* = 0.05 confidence level and were not adjusted for multiple comparisons. All analyses were performed using Stata/SE 15 (StataCorp).

### Reporting summary

Further information on research design is available in the Nature Research Reporting Summary linked to this article.

## Supplementary information

supplementary tables

Reporting Summary Checklist

## Data Availability

The data are not publicly available since they contain identifiable information that could compromise patient privacy.
