# Supplementary material for: Language impairment in adults with end-stage liver disease: application of natural language processing towards patient-generated health records
Source: NPJ Digit Med. 2019 Nov 4;2:106. doi: 10.1038/s41746-019-0179-9 (PMC6828779; doi:10.1038/s41746-019-0179-9)
Supplement: Supplementary file 1 — supplementary tables [file 41746_2019_179_MOESM1_ESM.pdf]

Supplementary Table 1: Transplant indications and matched controls

Transplant indication (n)	Matched control (n)	Alternative matched control (n)
Alcoholic cirrhosis (11)	Alcoholic liver disease (3)	Abnormal liver function tests (LFTs) (7), Hepatitis C virus (HCV)* (1)
Polycystic liver disease (3)	Polycystic liver disease (3)	
Budd-Chiari syndrome (1)	Budd-Chiari syndrome (1)	
Autoimmune cirrhosis (3)	Autoimmune hepatitis (3)	
Idiopathic cryptogenic cirrhosis (2), Wilson's disease (1)	Abnormal LFTs (3)	
Nonalcoholic steatohepatitis (NASH) cirrhosis (14)	Nonalcoholic steatohepatitis (14)	
HCV cirrhosis (27)	HCV (25)	Abnormal LFTs (2)
Crigler-Najjar syndrome (1)	Crigler-Najjar syndrome (1)	
Hepatic adenomatosis (1), Hepatocellcular carcinoma (2)	Benign neoplasm of the liver (3)	
Primary biliary cirrhosis (3)	Primary biliary cirrhosis (3)	
Primary sclerosing cholangitis (12)	Primary sclerosing cholangitis (9)	Abnormal LFTs (3)

*Secondary diagnosis for matched transplanted patient

Supplementary Table 2: Selected examples of paired pre-transplant and post-transplant messages

Pre-transplant messages	Post-transplant message
Good morning Dr. [Name], I am at Hopkins today for a couple of tests and an appointment with Dr. [Name], I have a couple of items I would like to ask you about. I have almost no energy and no real appetite food in general has no appeal.	Hey Doc, I understood someone from either your office or the study would call to schedule the appointments. It has been a while and I have not heard anything.
I'm up to 183+ thought we could up the water retention meds. Also four to five nights a week I'm waking throughout the night with searing pain in my chest I think its my gallbladder which I've considered going to the hospital for help.	[Name], have you read the MRI report? I have disc and report also. The report only is in my chart so you should also have it.
Dear my Dr, When the transplant team meets on Tuesday would you please call with an update on chemo results. [Name] said that the team might decide that chemo is good as it stands or might say do it again. Also I am going to send two papers that I need you to fill out, one has two sides for you to do the other one only has one side, I will scan them and attach them in this email.	Is this something I can do or do you suggest using a different doctor. Let me know if I can set up an appointment or not.
Hello Dr [Name], Just trying to follow up on my last prescription I went twice to the pharmacy, and the first time they were waiting on insurance approval, the second time I was told insurance did not cover it and the cost is $2000.	Good morning [Name]. Can you please check with Dr [Name] on the prescriptions from yesterday. I was able to pick up the powder, but not the water pills or ursodiol.
After disappointing results from using Aleve for my occasional headaches, which we discussed at our last appointment with you, I took advice from a friend who gets migraines, and tried CVD 315 mg which I purchased at my local natural foods store.	Hello Dr [Name]! I hope you are well. I have been experiencing profound pain in my right eye. It is infrequency and is a very sharp stabbing pain followed by pain in the socket.

Exclusion ICD-10 codes for cases and controls

F04	Amnestic Disorder Due To Known Physiological Condition
F09	Unspecified Mental Disorder Due To Known Physiological Condition
F03.90	Unspecified Dementia Without Behavioral Disturbance
G30.9	Alzheimer's Disease, Unspecified
G31.01	Pick's Disease
G31.09	Other Frontotemporal Dementia
G93.7	Reye's Syndrome
R41.0	Disorientation, Unspecified
R41.3	Other Amnesia
R47.01	Aphasia
R48.1	Agnosia
R48.2	Apraxia
G45.9	Transient Cerebral Ischemic Attack, Unspecified
I63.9	Cerebral Infarction, Unspecified
E72.00	Disorders Of Amino-Acid Transport, Unspecified
E72.01	Cystinuria
E72.09	Other Disorders Of Amino-Acid Transport
F70	Mild Intellectual Disabilities
F71	Moderate Intellectual Disabilities
F72	Severe Intellectual Disabilities
F73	Profound Intellectual Disabilities
F79	Unspecified Intellectual Disabilities
F84.0	Autistic Disorder
F84.3	Other Childhood Disintegrative Disorder
F84.5	Asperger's Syndrome
F84.8	Other Pervasive Developmental Disorders
Q87.89	Other Specified Congenital Malformation Syndromes, Not Elsewhere Classified
Q89.7	Multiple Congenital Malformations, Not Elsewhere Classified
Q89.8	Other Specified Congenital Malformations
Q89.9	Congenital Malformation, Unspecified
Q99.2	Fragile X Chromosome
R62.0	Delayed Milestone In Childhood
E70.0	Classical Phenylketonuria
E70.1	Other Hyperphenylalaninemias
E72.04	Cystinosis
E75.02	Tay-Sachs Disease
E75.249	Niemann-Pick Disease, Unspecified
E75.29	Other Sphingolipidosis
E76.29	Other Mucopolysaccharidoses

E76.3	Mucopolysaccharidosis, Unspecified
G03.9	Meningitis, Unspecified
G04.90	Encephalitis And Encephalomyelitis, Unspecified
G04.91	Myelitis, Unspecified
C71.6	Malignant Neoplasm Of Cerebellum
C71.9	Malignant Neoplasm Of Brain, Unspecified
C72.0	Malignant Neoplasm Of Spinal Cord
C75.1	Malignant Neoplasm Of Pituitary Gland
D32.9	Benign Neoplasm Of Meninges, Unspecified
D49.6	Neoplasm Of Unspecified Behavior Of Brain
Q85.00	Neurofibromatosis, Unspecified
G12.21	Amyotrophic Lateral Sclerosis
G12.29	Other Motor Neuron Disease
G10	Huntington's Disease
G20	Parkinson's Disease
G21.9	Secondary Parkinsonism, Unspecified
G23.1	Progressive Supranuclear Ophthalmoplegia
G71.0	Muscular Dystrophy
G71.11	Myotonic Muscular Dystrophy
G71.12	Myotonia Congenita
G71.14	Drug Induced Myotonia
G71.2	Congenital Myopathies
G72.2	Myopathy Due To Other Toxic Agents
G72.3	Periodic Paralysis
G11.1	Early-Onset Cerebellar Ataxia
G11.4	Hereditary Spastic Paraplegia
G35	Multiple Sclerosis
G36.0	Neuromyelitis Optica [Devic]
G80.9	Cerebral Palsy, Unspecified
G81.90	Hemiplegia, Unspecified Affecting Unspecified Side
G95.11	Acute Infarction
Q05.9	Spina Bifida, Unspecified
G12.9	Spinal Muscular Atrophy, Unspecified
G60.0	Hereditary Motor And Sensory Neuropathy
G60.1	Refsum's Disease
G61.0	Guillain-Barre Syndrome
G70.00	Myasthenia Gravis Without (Acute) Exacerbation
G70.01	Myasthenia Gravis With (Acute) Exacerbation
G11.8	Other Hereditary Ataxias
G12.29	Other Motor Neuron Disease
G12.8	Other Spinal Muscular Atrophies And Related Syndromes
G25.82	Stiff-Man Syndrome
G25.9	Extrapyramidal And Movement Disorder, Unspecified
G31.84	Mild Cognitive Impairment, So Stated
G31.9	Degenerative Disease Of Nervous System, Unspecified

G37.9	Demyelinating Disease Of Central Nervous System, Unspecified
G61.81	Chronic Inflammatory Demyelinating Polyneuritis
G70.00	Myasthenia Gravis Without (Acute) Exacerbation
G93.40	Encephalopathy, Unspecified
R41.2	Retrograde Amnesia
R41.89	Other Symptoms And Signs Involving Cognitive Functions And Awareness
R47.1	Dysarthria And Anarthria
R55	Syncope And Collapse
F84.5	Asperger's syndrome
F84.0	Autistic disorder
F31.81	Bipolar II disorder
F31.31	Bipolar disorder, current episode depressed, mild
F31.30	Bipolar disorder, current episode depressed, mild or moderate severity, unspecified
F31.32	Bipolar disorder, current episode depressed, moderate
F31.5	Bipolar disorder, current episode depressed, severe, with psychotic features
F31.4	Bipolar disorder, current episode depressed, severe, without psychotic features
F31.0	Bipolar disorder, current episode hypomanic
F31.2	Bipolar disorder, current episode manic severe with psychotic features
F31.11	Bipolar disorder, current episode manic without psychotic features, mild
F31.12	Bipolar disorder, current episode manic without psychotic features, moderate
F31.13	Bipolar disorder, current episode manic without psychotic features, severe
F31.10	Bipolar disorder, current episode manic without psychotic features, unspecified
F31.61	Bipolar disorder, current episode mixed, mild
F31.62	Bipolar disorder, current episode mixed, moderate
F31.64	Bipolar disorder, current episode mixed, severe, with psychotic features
F31.63	Bipolar disorder, current episode mixed, severe, without psychotic features
F31.60	Bipolar disorder, current episode mixed, unspecified
F20.2	Catatonic schizophrenia
F80.81	Childhood onset fluency disorder
F05	Delirium due to known physiological condition
F22	Delusional disorders
F02.81	Dementia in other diseases classified elsewhere with behavioral disturbance

F02.80	Dementia in other diseases classified elsewhere without behavioral disturbance
F48.1	Depersonalization-derealization syndrome
F20.1	Disorganized schizophrenia
F44.0	Dissociative amnesia
F44.9	Dissociative and conversion disorder, unspecified
F44.1	Dissociative fugue
F44.81	Dissociative identity disorder
F44.2	Dissociative stupor
F20.0	Paranoid schizophrenia
F84.9	Pervasive developmental disorder, unspecified
F48.2	Pseudobulbar affect
F06.2	Psychotic disorder with delusions due to known physiological condition
F06.0	Psychotic disorder with hallucinations due to known physiological condition
F25.0	Schizoaffective disorder, bipolar type
F25.1	Schizoaffective disorder, depressive type
F25.9	Schizoaffective disorder, unspecified
F20.81	Schizophreniform disorder
F03.91	Unspecified dementia with behavioral disturbance
F03.90	Unspecified dementia without behavioral disturbance
F89	Unspecified disorder of psychological development
F09	Unspecified mental disorder due to known physiological condition
F01.51	Vascular dementia with behavioral disturbance
F01.50	Vascular dementia without behavioral disturbance
